# PReS-FINAL-2229: Pamidronate in CRMO - a small case series

**DOI:** 10.1186/1546-0096-11-S2-P219

**Published:** 2013-12-05

**Authors:** M Schuller, J Franova, M Macku

**Affiliations:** 1 University Hospital Brno, Brno, Czech Republic

## Introduction

Chronic recurrent multifocal osteomyelitis (CRMO) is a rare autoinflammatory disorder which affects predominantly girls with peak onset between ages of 7 to 12 years. There is a frequent association of CRMO with inflammatory skin or gut disorders. Patients with isolated CRMO are usually treated with nonsteroidal anti-inflammatory drugs (NSAIDs) prior to escalation to corticoids and disease-modifying antirheumatic drugs such as methotrexate (MTX), sulfasalazine, azathioprine. Recently, TNF inhibitors and bisphosphonates have been recommended for treatment of most severe cases.

## Objectives

To evaluate pamidronate treatment in patients with isolated CRMO relapsing despite NSAID, corticosteroid and methotrexate therapy in a retrospective study of a case series.

## Methods

Since 2011, 4 patients (3 girls and 1 boy) with CRMO have been treated with pamidronate in Paediatric Department of University Hospital Brno, Czech Republic. All these patients had chronic relapsing multifocal osteomyelitis without any associated inflammatory condition. The diagnosis of CRMO was set in the mean age of 12 years based on radiographic findings (x-ray, CT, MRI) and histological findings of a non-bacterial inflammatory bone lesion. Previous treatment with NSAIDs, corticosteroids, and MTX (MTX used only in 3 of 4 cases) was insufficient.

## Results

Intravenous pamidronate administered every 3 to 6 months was added to MTX in 3 patients, in the fourth case it was started in a MTX naive patient. Corticosteroids were used to control acute symptoms. All the patients with pamidronate significantly improved. In 3 patients including 1 patient without MTX no corticosteroids were needed after 1 month of pamidronate therapy and there are no clinical signs of the disease activity now. In 1 patient treated with pamidronate and MTX the dose of corticosteroids has significantly decreased. No adverse event was observed.

## Conclusion

In accordance with previous observations of other authors the results of our small case series indicate good efficacy of pamidronate treatment in patients with CRMO. In contrast, MTX alone had no benefit in our patients. We recommend considering pamidronate a second line therapy in more severe cases of isolated CRMO.

## Disclosure of interest

None declared.

